# Human serum influences functional plasticity and transcriptomic landscape of γδ T cells *in vitro*

**DOI:** 10.3389/fimmu.2026.1722590

**Published:** 2026-02-10

**Authors:** Lorraine Pinot, Zhibek Zhumadilova, Aylin Saßor, Sagar, José Villacorta Hidalgo

**Affiliations:** 1 Research and Development Immunotherapy, Miltenyi Biotec, Bergisch Gladbach, Germany; 2 Department of Immunology, Eberhard Karls Universität Tübingen, Tübingen, Germany; 3 Department of Medicine II (Gastroenterology, Hepatology, Endocrinology, and Infectious Diseases), Freiburg University Medical Center, Faculty of Medicine, University of Freiburg, Freiburg, Germany; 4 Faculty of Biology, University of Freiburg, Freiburg, Germany

**Keywords:** allogeneic, gamma-delta T cells, immunotherapy, serum-free medium, single-cell RNA sequencing

## Abstract

γδ T cells are emerging as a promising platform for adoptive cell therapy due to their ability to recognize tumors independently of MHC and their minimal risk of causing graft-versus-host disease. While serum-supplemented media have traditionally been used for T cell expansion, they can present limitations including xenogeneic contaminants and batch variability. These issues can compromise T cell phenotype, function, and clinical reproducibility. In this study, we evaluated the impact of human serum on the expansion, phenotype, function, and transcriptomic landscape of Vγ9Vδ2 γδ T cells cultured with zoledronate and cytokines under serum-free versus serum-containing conditions. We evaluated cytotoxicity against triple-negative breast cancer cell lines, activation and checkpoint marker expression, and cytokine secretion. Single-cell RNA and TCR sequencing revealed consistent differentiation trajectories across donors in both conditions and gene expression dynamics during a two-week expansion period. Our results show that serum-free culture supports robust γδ T cell expansion with higher purity and an activated phenotype marked by increased activation markers and reduced checkpoint receptor expression. Serum-free expanded cells displayed comparable or enhanced cytotoxicity and cytokine production, especially IFN-γ. TCR repertoire diversity was preserved without clonal skewing in both conditions. Furthermore, re-exposure to serum late in culture had minimal influence on γδ T cell functionality. These findings demonstrate the feasibility and advantages of serum-free expansion protocols for Vγ9Vδ2 γδ T cells, offering improved consistency, safety, and therapeutic potential.

## 1 Introduction

Gamma delta (γδ) T cells possess distinctive functional capacities that position them as a promising cell platform for adoptive cell therapy. γδ T cells can recognize and kill tumor cells independently of major histocompatibility complex (MHC), thereby circumventing a common mechanism of tumor immune evasion. This MHC-unrestricted recognition enables γδ T lymphocytes to detect stressed, infected or transformed cells across a broad spectrum of malignancies (1–3). This intrinsic reactivity, combined with a minimal risk of Graft versus Host Disease (GvHD) is particularly interesting in allogeneic settings. Furthermore, γδ T cells possess an inherent capacity to infiltrate solid tumors, including highly aggressive subtypes (4–6). The successful application of γδ T cells in cancer immunotherapy critically depends on optimizing their ex-vivo expansion to achieve sufficient cell numbers with an appropriate phenotype required for therapeutic efficacy (7, 8).

In recent years, zoledronate, a third-generation amino-bisphosphonate has become a pivotal agent for the efficient ex-vivo expansion of Vγ9Vδ2 T cells. Zoledronate indirectly activates Vγ9Vδ2 T cells by inhibiting the farnesyl pyrophosphate synthase (FPPS) in the mevalonate synthesis pathway. This inhibition results in the intracellular accumulation of isopentenyl pyrophosphate (IPP), a phosphoantigen that is specifically recognized by Vγ9Vδ2 T cells, facilitated by butyrophilin family proteins (9–11). This method enables large-scale expansion of functional γδ T cells facilitating their potential therapeutic use.

Serum-supplemented media have long been the standard for T cell expansion but introduce some limitations including xenogeneic contaminants, batch variability and a risk of microbial contamination. These issues can compromise T cell phenotype, function, and clinical reproducibility (12). To address these concerns, regulatory agencies such as the FDA (13) and EMA (14) advocate for serum-free media in cell-based therapies to ensure greater safety, consistency, and reproducibility. Moreover, serum-free conditions have been shown to support the expansion and function of engineered αβ T cells (15, 16).

Although most current expansion protocols for γδ T cells, including those used clinically, rely on human serum supplementation (3, 17–21) γδ T cells can be efficiently expanded under serum-free conditions upon stimulation with zoledronate and cytokines (22). However, both their expansion and functionality are highly sensitive to culture medium composition (23). The specific impact of these media formulations on Vγ9Vδ2 T cell functionality remains poorly defined, while ex-vivo-expanded γδ T cells have rarely been studied using single-cell multiomics.

In this study, we investigate how the presence of human serum in culture influences γδ T cells and evaluate its implications for adoptive cell therapy. We compare the expansion, phenotype, and function of γδ T cells cultured in serum-free versus serum-containing media. To elucidate the mechanisms underlying enhanced functionality in expanded γδ T cells, we apply time-course single-cell multiomics to profile transcriptomes and TCR repertoires. By comprehensively characterizing these changes, our work aims to support the development of safer and more effective immunotherapies based on γδ T cells. Optimizing culture conditions for γδ T cell expansion may help the clinical translation of this approach and improve therapeutic outcomes for cancer patients.

## 2 Materials and methods

### 2.1 Antibodies and reagents

The following antibodies were used for cell surface staining and intracellular staining: anti-human CD107a REA803, anti-human CD14 REA599, anti-human CD19 REA675, anti-human CD27 REA499, anti-human CD3 REA613, anti-human CD45RA REA562, anti-human CD56 REA196, anti-human CD69 REA824, anti-human Granzyme B REA226, anti-human HLA/DR REA805, anti-human IFNγ 45-15, anti-human KIR2D REA1042, anti-human NKG2D REA797, anti-human PD-1 REA1165, anti-human Perforin REA1061, anti-human REA Control (I) REA293, anti-human REA Control (S) REA293, anti-human TCR Vδ1 REA173, anti-human TCR Vδ2 REA771, anti-human TCR γδ REA591, anti-human TIGIT REA1004, and anti-human TIM3 F38-2E2 (all from Miltenyi Biotec). PEB buffer was prepared by adding 0.5% HSA (Octapharma) in CliniMACS buffer (Miltenyi Biotec).

### 2.2 Tumor cell lines

Human triple-negative breast cancer (TNBC) cell lines expressing GFP and luciferase were used in this study, including MDA-MB-231, MDA-MB-468, and MDA-MB-157. The wild-type cells were obtained from the Leibniz Institute DMSZ and were then transduced with a GFP-P2A-Luc plasmid using a VSV-G LV. They were cultured in Dulbecco’s Modified Eagle Medium (DMEM) supplemented with glutamine and sodium pyruvate (Biowest), 10% fetal bovine serum (FBS) (Biowest), and 100mM MEM non-essential amino acids (Gibco). The cells were maintained in a humidified incubator at 37°C with 5% CO_2_.

### 2.3 γδ T cell expansion

Peripheral blood mononuclear cells (PBMCs) were isolated from whole blood donations. Isolation was performed using density gradient centrifugation with Pancoll (PAN-Biotech), following the manufacturer’s instructions.

The cells were then plated at 2 x 10^6^ cells/ml in TexMACS medium (Miltenyi Biotec). The medium was supplemented with 100 IU/ml interleukin-2 (IL-2) (Miltenyi Biotec), 100 IU/ml interleukin-15 (IL-15) (Miltenyi Biotec), and 5µM Zoledronate (Zometa^®^ Roche). For samples containing serum, 5% human AB serum (Access Biologicals) was added to the culture medium. Samples initially cultured without human serum were subsequently re-exposed to 5% serum to simulate the conditions encountered during allogeneic interventions. The cultures were maintained in a humidified incubator at 37°C with 5% CO_2_. Medium and cytokines were refreshed every 2–3 days to support cell growth and expansion.

### 2.4 Phenotyping and cell surface markers analysis

At different time points during the γδ T cell expansion, the cells were harvested and washed with PEB buffer. For phenotyping and cell surface marker analysis, the expanded γδ T cells were stained with specific antibodies, as mentioned in the “Antibodies and Reagents” section and in **Supplementary Figure S1**, and with 7-AAD (Miltenyi Biotec) to identify dead cells. In particular, CD45RA and CD27 antibodies were used to identify and characterize the γδ T cell phenotype. To minimize non-specific binding, FcR-blocking reagent was added to the staining mix (Miltenyi Biotec). The staining process was performed at 4°C for 10 minutes. Subsequently, the cells were washed twice with PEB. The stained cells were then analyzed using a MACSQuant 10 flow cytometer (Miltenyi Biotec). The flow cytometry data were analyzed using FlowJo software (BD Bioscience). The gating strategy is shown in **Supplementary Figure S2**.

### 2.5 *In vitro* assay for γδ T cell cytotoxicity

To specifically assess γδ T cells, αβ T cells were efficiently depleted from PBMCs using magnetic separation prior to expansion. Briefly, PBMCs were resuspended in phosphate-buffered saline with 0.5% bovine serum albumin (PEB) and incubated with anti-human TCR αβ-biotin antibodies for 30 minutes at room temperature. After two washes with PEB, the cells were incubated with anti-biotin microbeads (Miltenyi Biotec) for an additional 30 minutes at room temperature. Subsequently, the cells were washed once and resuspended in PEB before being passed through an LS column (Miltenyi Biotec) on a magnetic separator. The unbound fraction, enriched with γδ T cells, was collected for further expansion, as described above.

γδ T cell cytotoxicity was assessed with two complementary methods: the luciferase assay and the Incucyte system (Sartorius). In the luciferase assay, γδ T cells were co-cultured with luciferase-expressing target cells at various effector to target (E:T) ratios for 24 hours to measure cytotoxic activity. Target cell viability was determined by quantifying luminescence using the One-Glo luciferase assay kit (Promega) and measuring it with a Victor 3 plate reader (Perkin Elmer). Additionally, for real-time cytotoxicity monitoring, GFP-expressing target cells were co-cultured with γδ T cells at different E:T ratios in the Incucyte system, which tracked GFP+ target cell area confluence every 2 hours for several days.

### 2.6 γδ T cells degranulation, cytokine production and secretion assays

Three distinct assays were employed to assess the cytotoxic granule-mediated killing and cytokine secretion capabilities of γδ T cells. The CD107a degranulation assay involved coculturing γδ T cells with target cells for 2 hours in a humidified incubator at 37°C with 5% CO_2_, anti-CD107 antibody (Miltenyi Biotec) and Bafilomycin A1 (Sigma). The cells were then stained with anti-7AAD, anti-CD3, and anti-TCRγδ antibodies and analyzed using a MACSQuant flow cytometer, with data analysis conducted through FlowJo software. For intracellular cytokine staining, γδ T cells were cocultured with target cells for 2 hours in the same incubation conditions, but in the presence of Brefeldin A (Sigma). Subsequently, live/dead staining was performed using Viobility 405/520 fixable dye (Miltenyi Biotec), followed by cell fixation with Inside Fix (Miltenyi Biotec) and permeabilization with Inside Perm (Miltenyi Biotec). The cells were then stained with anti-CD3, anti-TCRγδ, IFN-γ, Granzyme B (GzmB), TNFα and Perforin (PFN) antibodies, washed twice, and analyzed with the MACSQuant and FlowJo software. Additionally, for cytokine secretion assessment, we collected supernatants after 24 hours of coculture between γδ T cells and target cells. The frozen supernatant was processed using the MACSPlex T/NK assay (Miltenyi Biotec) following the manufacturer’s instructions, allowing simultaneous measurement of multiple cytokines. The data was only considered when the target cells were not secreting any measurable amount of a cytokine.

### 2.7 Single-cell simultaneous gene expression and TCR profiling

γδ T cells were frozen at different time points using MACS formulation solution (Miltenyi Biotec), a serum-free cryopreservation medium. After thawing, the cells were washed with PBS and stained with mouse anti-human TCRγ/δ IgG1κ PE (Miltenyi Biotec, 130-113-504; dilution 1:200) and 4′,6-diamidino-2-phenylindole (DAPI) at a final concentration of 30 µM. TCRγ/δ^+^ DAPI^-^ cells were sorted into 2% BSA-coated 1.5 ml Eppendorf tubes. Single-cell RNA sequencing (scRNA-seq) was performed using the 10x Genomics platform with feature barcoding technology, enabling multiplexing of samples from different conditions to reduce cost and minimize technical variability. Samples from three donors were included in the analysis. For each donor, seven conditions (day 0; day 7, 10, and 14 with and without serum) were multiplexed using TotalSeq-C hashtag antibodies listed in **Supplementary Figure S3** (BioLegend) except day 0 sample from donor A which was separately processed. Barcoded antibodies were applied during the staining step prior to FACS sorting, at a concentration of 1 µg per million cells, as recommended by the manufacturer. After staining, cells were washed twice in PBS containing 2% BSA and 0.01% Tween 20, followed by centrifugation (400 × g, 5 minutes, 4°C) and supernatant exchange. Cells were then resuspended in PBS, filtered through 40 µm cell strainers, and prepared for sorting. Sorted γδ T cells were processed using the 10x Genomics single-cell 5′ V(D)J workflow according to the manufacturer’s instructions. For each donor, 30,000 cells representing the seven conditions were pooled and loaded into a single 10x Genomics reaction. TCR libraries were generated using the following primers – First PCR: 1 µM forward primer (5′-GATCTACACTCTTTCCCTACACGACGC-3′) and 1 µM of each reverse primer (5′-ATCCCAGAATCGTGTTGCTC-3′ and 5′-CCCACTGGGAGAGATGACAA-3′) and Second PCR: 1 µM forward primer (5′-GATCTACACTCTTTCCCTACACGACGC-3′) and 1 µM of each reverse primer (5′-GGGGAAACATCTGCATCAAG-3′ and 5′-GACAAAAACGGATGGTTTGG-3′) (24). Libraries were pooled in appropriate proportions to achieve the desired sequencing depth, as recommended by 10x Genomics, and sequenced using a NovaSeq 6000 flow cell.

### 2.8 Single-cell computational data analysis

Sequencing reads were processed using CellRanger’s (v6.0.0) count pipeline to quantify gene expression and antibody-derived hashtag counts. Alignment was performed with the pre-built GRCh38-2020-A reference. TCR sequencing data were analyzed with MiXCR’s analyze 10x-sc-xcr-vdj wrapper function, leveraging the built-in reference library for annotation (25). scRNA-seq count matrices were processed and analyzed in R (v4.2.3 and v4.5.0) using the Seurat package (v4.3.0 and v5.3.0) (26). Hashtag counts were normalized (normalization.method = “CLR”) and used for demultiplexing with the HTODemux function. Cells identified as singlets were retained for downstream analyses. Low-quality cells were excluded based on the number of detected genes and the percentage of mitochondrial genes (specific thresholds for each donor). To remove batch effects, we integrated the data using Harmony (27). Harmony was executed using the RunHarmony function in Seurat with group.by.vars set to each donor. Importantly, ribosomal genes (small and large subunits) as well as mitochondrial genes with MT- identifier were excluded from the analysis. The normalization method was set to ‘LogNormalize’. Dimensionality reduction was performed using the RunUMAP function, where reduction was set to ‘harmony’ and dims to 1:30. Default resolution was used for clustering. To characterize the clusters, differential gene expression analysis was performed using the FindMarkers function in Seurat. TCR repertoire data were processed, analyzed, and integrated with scRNA-seq data using the scRepertoire package (v2.0.8). While performing the TCR repertoire analysis using scRepertoire, cloneCall parameter was set to ‘strict’, which uses the V(D)JC genes comprising the TCR plus the nucleotide sequence of the CDR3 region to call the clonotypes. Both γ and δ chains were used for the clonotype analysis wherever detected. Shannon diversity score was calculated based on amino acid sequences of the TRG repertoire.

### 2.9 Statistical analysis

Flow cytometry and cytotoxicty data were analyzed with GraphPad Prism (Dotmatics). Šídák’s multiple comparisons test with paired data were used unless stated otherwise, in which case paired Wilcoxon tests were used. A p-value below 0.05 was considered significant. For scRNA-seq data, differential gene expression between clusters or disease states was computed using Seurat’s FindMarkers function (Wilcoxon rank-sum test, adjusted by Bonferroni correction). Statistical details for each analysis are provided in the figure legends.

### 2.10 Data and code availability

Sequencing data (FASTQ files) are available upon request to S. The processed data reported in this paper is available to download from https://doi.org/10.5281/zenodo.17590095. Codes to reproduce the data analysis and figures are available at the same repository.

## 3 Results

### 3.1 Expansion of Vγ9Vδ2 T cells in serum-free conditions alters activation and checkpoint marker expression without affecting proliferation

**Figure 1:**
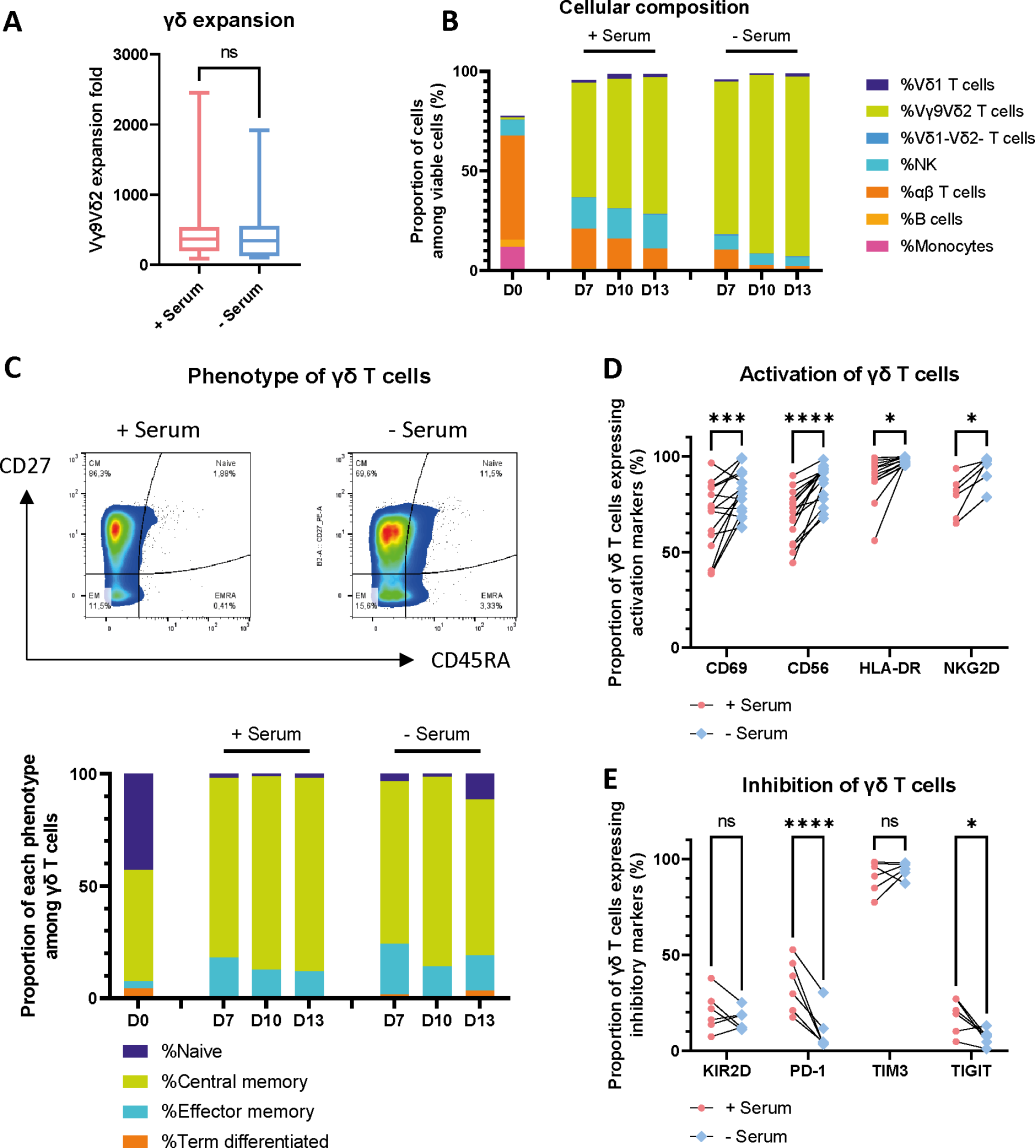
Impact of serum on γδ T cell expansion and phenotype. **(A)** Fold expansion comparison with and without 5% human AB serum in the culture medium, analyzed using the Wilcoxon matched pairs signed rank test, n=14. **(B)** Cellular composition assessed by flow cytometry throughout the expansion, illustrated for a representative donor. **(C)** γδ T cell phenotype determined by flow cytometry based on CD27 and CD45RA expression. Exemplary plots on day 13 and proportions throughout the culture for a representative donor. **(D)** Expression of activation markers on γδ T cells analyzed by flow cytometry. Šídák’s multiple comparisons test, n=15 for CD69, CD56, and HLA-DR; n=6 for NKG2D. **(E)** Expression of inhibitory and exhaustion markers on γδ T cells, analyzed by flow cytometry. Šídák’s multiple comparisons test, n=15. In the figures, the significance levels denoted by stars are as follows: *p < 0.05, ***p < 0.001, ****p < 0.0001, ns = non-significant.

In this study, we investigated the impact of human serum supplementation on Vγ9Vδ2 T cell expansion and phenotypic differentiation using a standard method with zoledronate and low dose of IL-2 and IL-15. Our data demonstrates that the absolute expansion of γδ T cells did not significantly differ between serum-containing and serum-free culture conditions (p=0.4631 in a paired Wilcoxon test, **Figure 1A**, n=14); γδ T cells expanded 526.8 folds, 95% CI [170.1 – 883.4] in media supplemented with serum, and 462.0 folds, 95% CI [183.9 – 740.1] in serum-free conditions. Post-expansion, flow cytometric analysis revealed a marked enrichment of Vγ9Vδ2 T cells (p=0.0002 in a paired Wilcoxon test, **Supplementary Figure S4A**, n=12) concomitant with a significant reduction in NK cell populations ((p=0.0039 in a paired Wilcoxon test, **Supplementary Figure S4B**, n=12) (**Figure 1B**). The purity at harvest was 71.65%, 95% CI [60.72 – 82.58] with serum and 86.78%, 95% CI [83.53 – 90.03] in serum-free conditions and without prior αβ depletion (n=10), cell viability remained high (**Supplementary Figure S5**), thus establishing serum-independence for achieving Vγ9Vδ2 T cell proliferation with this method and suggesting a selective enrichment of γδ T cells during the ex-vivo expansion process.

Phenotypic characterization of the expanded γδ T cells indicated a predominant memory phenotype, with over 80% expressing CD27 in 10 out of 12 donors (**Figure 1C**). Interestingly, no statistically significant differences in memory phenotype distribution were observed between the serum-containing and serum-free expanded cohorts except for a small decrease in central memory cells in serum-free conditions (p=0.0110, **Supplementary Figure S6**), suggesting that the presence or absence of serum does not critically influence the cell product phenotype. Furthermore, a detailed assessment of activation and inhibitory receptor expression revealed an impact on these markers. Cells expanded in serum-free conditions exhibited significantly upregulated surface expression of activation markers, including CD69 (p=0.0003, n=15), CD56 (p<0.0001, n=15), HLA-DR (p=0.0242, n=15) and NKG2D (p= 0.0225, n=6), relative to their serum-supplemented counterparts (**Figure 1D**, confidence intervals are reported in **Supplementary Figure S7**). We next evaluated the expression of immune checkpoint and inhibitory receptors on γδ T cells; while KIR2D and TIM3 expression remained unaffected by serum absence, a notable decrease in the expression of PD-1 (p<0.0001) and TIGIT (p=0.0363) was observed (**Figure 1E**, n=6, confidence intervals are reported in **Supplementary Figure S7**). Taken together, these results indicate that robust and reproducible proliferation rate of Vγ9Vδ2 T cells can be achieved independently of serum supplementation in the culture medium. However, while the proliferative capacity and memory subset composition remain largely unaltered, serum deprivation during ex-vivo expansion induces distinct modulation of key activation and inhibitory receptor expression.

### 3.2 Serum in culture modulates CD107a-mediated degranulation without significantly affecting the cytotoxicity of activated γδ T cells against TNBC cell lines

To further elucidate the role of serum in modulating the cytotoxicity against tumor cells, we evaluated the killing capacity of activated γδ T cells in the presence and absence of serum. After two weeks of expansion, we investigated the cytotoxicity of γδ T cells against triple-negative breast cancer (TNBC) cell lines, a tumor where γδ T cells have shown a reactive infiltration (28).

**Figure 2:**
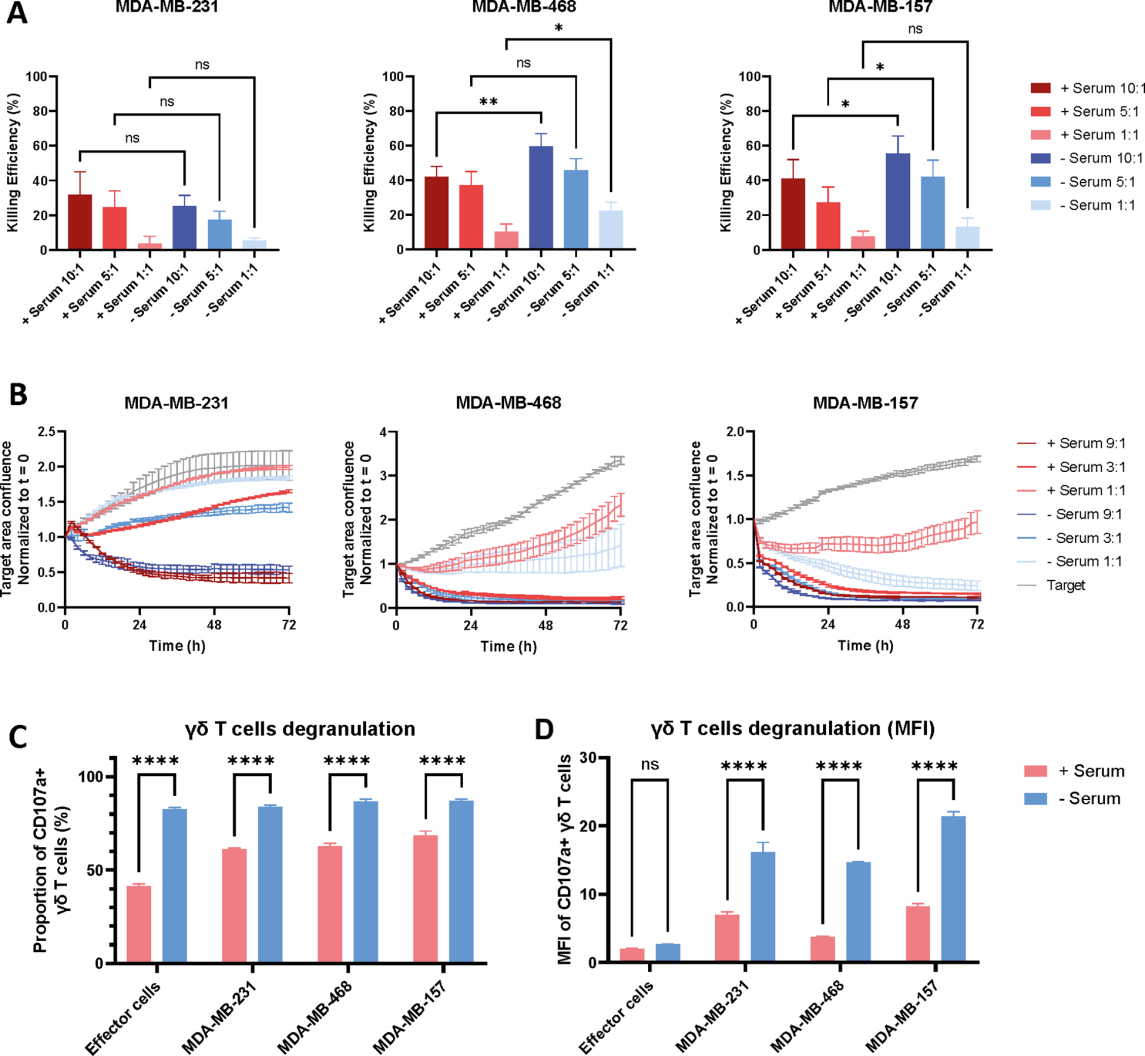
Impact of serum on γδ T cell cytotoxicity. γδ T cells were co-cultured with GFP and luciferase-expressing triple-negative breast cancer cell lines MDA-MB-231, MDA-MB-468, or MDA-MB-157 to assess their functionality. **(A)** Luciferase-based assay at different E:T ratios after 24h co-culture. Šídák’s multiple comparisons test (only similar E:T ratio comparisons are shown). n=5 for MDA-MB-231, n=9 for MDA-MB-468, n=8 for MDA-MB-157. **(B)** Incucyte assay tracking the confluence of GFP+ target cells over time at different E:T ratios, presented for one representative donor. **(C, D)** Measurement of CD107a expression on γδ T cells alone (effector cells) or in co-culture for 2h. Both the proportion of CD107a+ γδ T cells **(C)** and the MFI of this population **(D)** are shown. Šídák’s multiple comparisons test, one representative donor. In the figures, the significance levels denoted by stars are as follows: *p < 0.05, **p < 0.01, ****p < 0.0001, ns = non-significant.

We evaluated the cytotoxicity using a luciferase-based cytotoxic assay and the Incucyte live-cell imaging system. Both assays demonstrated that activated γδ T cells exert potent cytotoxic effects against the three TNBC cell lines MDA-MB-231, MDA-MB-468, and MDA-MB-157. Notably, MDA-MB-231 cells exhibited comparatively greater resistance to γδ T cell-mediated killing than the other two cell lines. Remarkably, in serum-free conditions, the cytotoxic activity was maintained or even enhanced across all effector-to-target (E:T) ratios relative to serum-containing conditions. (**Figures 2A, B**, for each cancer cell line: n=5, n=9 and n=8 respectively).

Given the key role of degranulation as mechanism underlying γδ T cell-mediated cytotoxicity, we evaluated CD107a expression by flow cytometry. Notably, a substantial proportion of γδ T cells exhibited CD107a expression even in the absence of target cells. Furthermore, the presence of serum significantly enhanced CD107a expression across all experimental conditions, including effector cells alone and co-cultures with the three triple-negative breast cancer cell lines: MDA-MB-231, MDA-MB-468, and MDA-MB-157 (p<0.0001 for each target cell line, **Figure 2C**, n=3).

To further elucidate degranulation dynamics, we quantified the median fluorescence intensity (MFI) of CD107a expression (**Figure 2D**, n=3). The MFI was significantly elevated in the co-culture conditions (p<0.0001), indicating enhanced degranulation of γδ T cells upon direct contact with target cells. Notably, the difference in CD107a MFI between serum-containing and serum-free conditions reached statistical significance only in the co-culture groups (p=0.5538 without target cells and p<0.0001 for each target cell line). These results underscore the robust cytotoxic and degranulation capacity of activated γδ T cells against the TNBC cell lines. Moreover, the presence of serum appears to specifically potentiate degranulation activity during γδ T cell–target cell interactions.

### 3.3 Single-cell transcriptomic and TCR profiling reveals distinct differentiation trajectories of γδ T cells during expansion with and without serum

**Figure 3:**
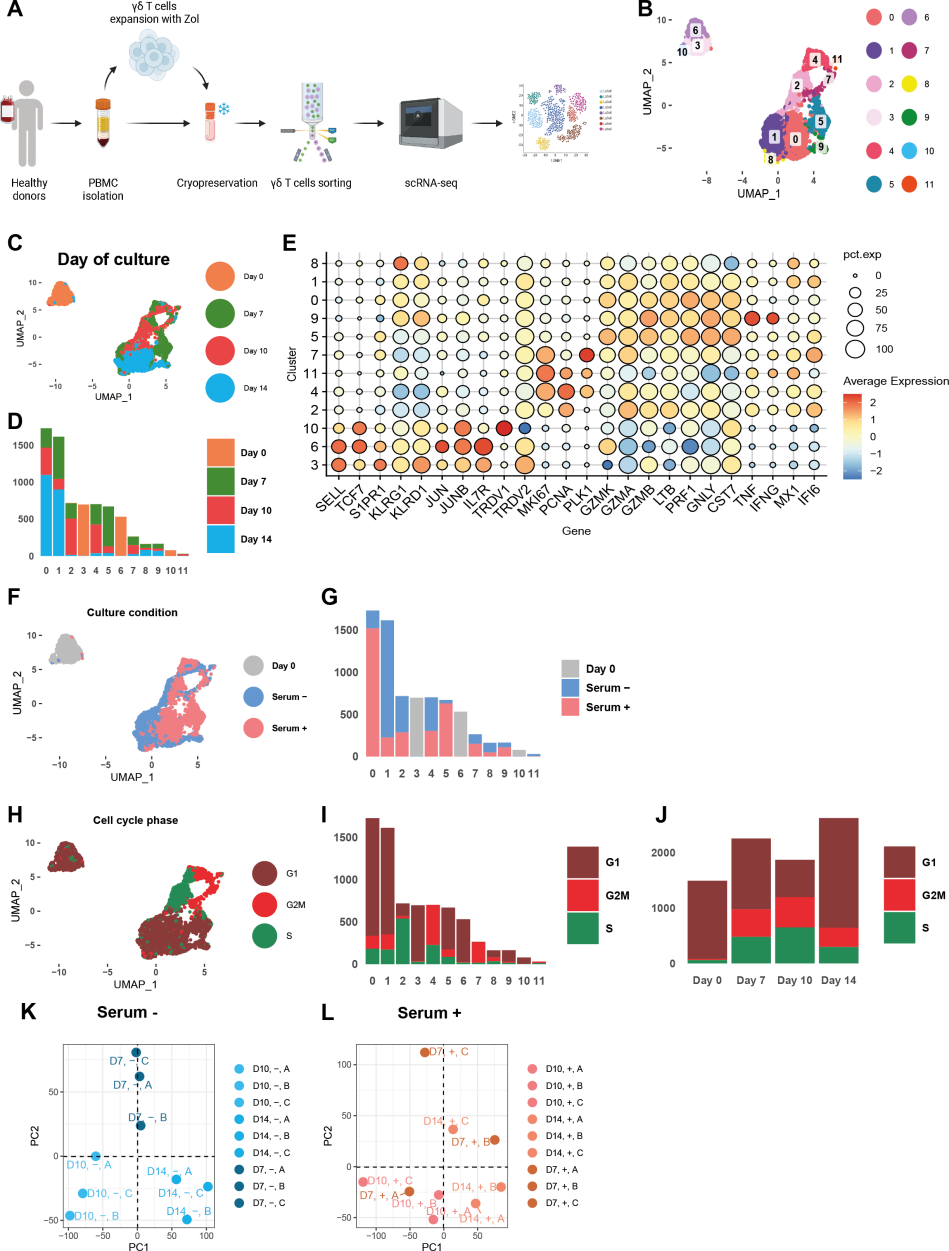
Single-cell transcriptomic profiling demonstrates robust and consistent γδ T cell differentiation in serum-free culture across donors and time. **(A)** Schematic representation of the experimental workflow. γδ T cells were isolated from three healthy donors, cultured under serum-free and with serum conditions, and sampled at multiple timepoints for single-cell RNA sequencing analysis. **(B)** UMAP projection of 8,249 γδ T cells from all donors, colored by unsupervised clustering, identifies 12 transcriptionally distinct cell clusters (clusters 0–11). **(C)** UMAP colored by day of culture (days 0, 7, 10, and 14). **(D)** Bar plots quantifying the number of cells from each culture day within each cluster. **(E)** Dot plot of key cluster-defining genes. Color intensity indicates mean expression within each cluster, and dot size reflects the proportion of cells expressing the gene. **(F)** UMAP projection colored by condition, cultured with or without serum in different colors. **(G)** Bar plots displaying the number of cells from each condition within each cluster. **(H)** UMAP colored by cell cycle phase, visualizing proliferative states across clusters in different colors. **(I)** Bar plots showing the distribution of cell cycle phases within each cluster. **(J)** Bar plots showing the distribution of cell cycle phases within each day of harvest. **(K, L)** Principal component analysis (PCA) of average gene expression across timepoints in serum-free and serum-containing cultures.

To assess transcriptional changes during γδ T cell expansion in both conditions, we performed single-cell profiling, including transcriptome and TCR repertoire, at days 0, 7, 10, and 14 of culture across three donors (**Figure 3A**; **Supplementary Figure S8**). Unsupervised clustering of scRNA-seq data identified 12 distinct cell clusters (**Figure 3B**). Cells from day 0 were largely restricted to clusters 3, 6, and 10 (**Figures 3C, D**). Clusters 3 and 6 expressed naïve and migratory markers (e.g., *TCF7*, *IL7R*, *SELL*, *S1PR1*), and were Vδ2^+^ (*TRDV2*), consistent with the typical composition of peripheral blood samples (**Figure 3E**). Cluster 3 also showed high expression of cytotoxic genes (*GZMB*, *GNLY*, *PRF1*), whereas cluster 6 had lower cytotoxicity and elevated *GZMK* (**Figure 3E**). Cluster 10 was Vδ1^+^ (*TRDV1*) and primarily expressed naïve-associated genes (**Figure 3E**). Cells from days 7 and 10 from serum-supplemented and serum-free cultures populated clusters 2, 4, 7, and 11 (**Figures 3D, F, G**). These were proliferative, marked by *MKI67* expression (**Figure 3E**). Cell cycle analysis revealed cluster 2 enriched for S-phase, cluster 4 for both S and G2/M phases, and cluster 7 mainly in G2/M (**Figures 3H, I, J**). By day 14, most cells converged into clusters 0 (containing serum-supplemented cells) and 1 (mainly comprised of serum-free cells), characterized by cytotoxic effector profiles (*GZMB*, *PRF1*, *GNLY*) and loss of naïve markers (**Figures 3E, F, G**).

To assess donor consistency, we performed PCA on average gene expression across timepoints. In serum-free cultures, samples from different donors aligned along a clear trajectory, with principal components separating days 7, 10, and 14 (**Figure 3K**). In contrast, serum-supplemented cultures showed high variability and lacked temporal structure (**Figure 3L**). These findings suggest that serum-free culture enables more uniform and reproducible γδ T cell differentiation across donors.

### 3.4 Serum influences temporal expression of interferon and cytotoxic genes in expanding γδ T Cells

**Figure 4:**
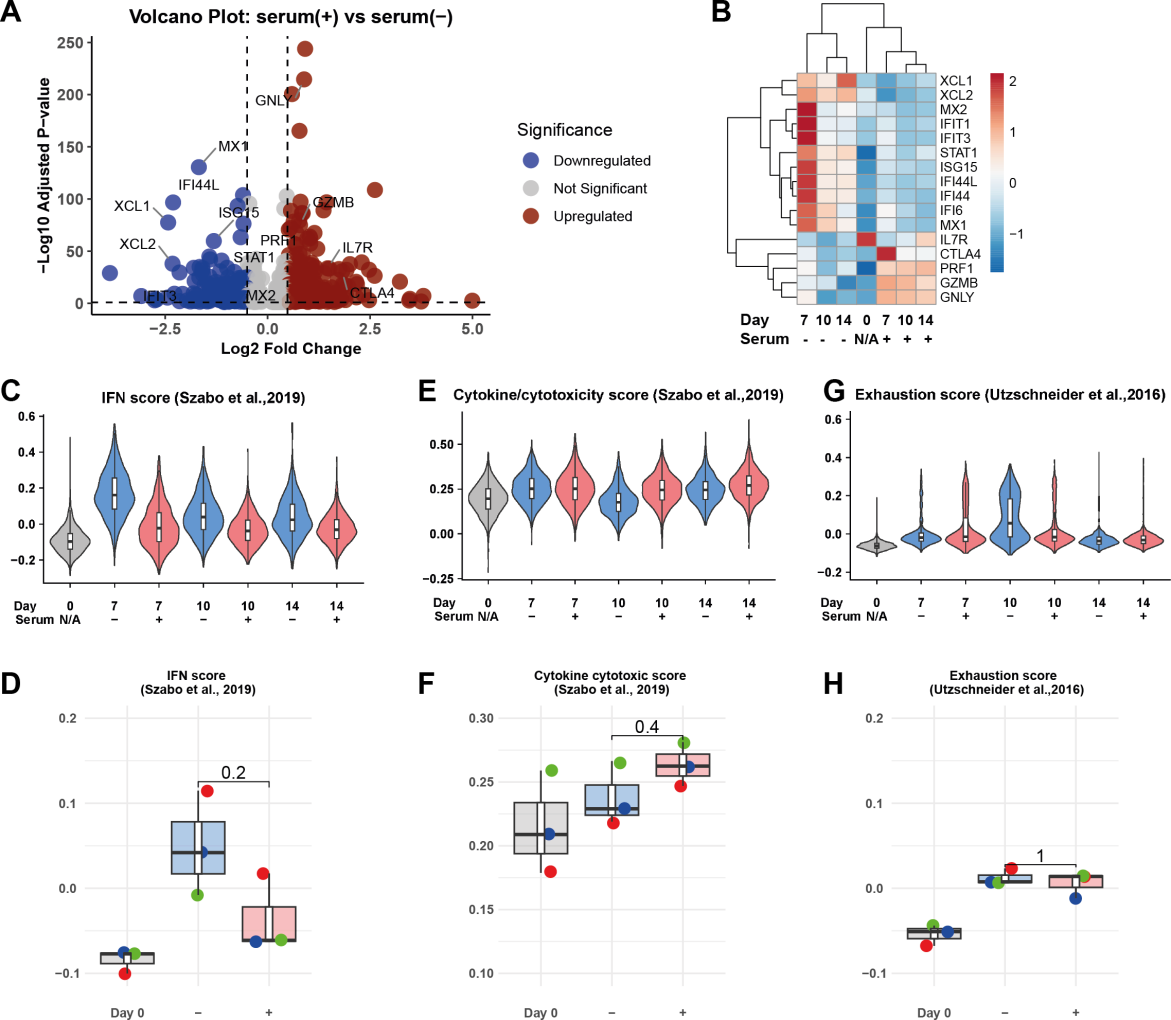
Serum-free culture Serum-free culture modulates effector programs at the single-cell transcriptomic level modulates effector programs at the single-cell transcriptomic level. **(A)** Volcano plots display differential gene expression between serum-free and serum-containing conditions, highlighting significant upregulation of interferon-related genes in serum-free cultures (log2 fold change vs. log10 adjusted p-value). **(B)** Heatmap of key cytotoxic and interferon-associated genes across days 0, 7, 10, and 14, comparing serum-free and serum-supplemented conditions. **(C, E, G)** Violin plots illustrate IFN, cytokine-cytotoxicity and exhaustion module scores across days of incubation and serum conditions. **(D, F, H)** Boxplots summarize module scores for each donor (n=3), with each point representing an independent donor. Statistical significance was determined using the Wilcoxon test.

To quantify the transcriptional differences between serum and serum-free conditions on a genome-wide scale, we performed differential gene expression analysis on pooled γδ T cells from Day 7, 10, and 14 cultures. Notably, serum-supplemented conditions were associated with increased expression of cytotoxic genes such as *GZMB*, *GNLY*, and *PRF1* (**Figure 4A**). In contrast, serum-free conditions showed upregulation of interferon-stimulated genes (ISGs), including *MX1*, *MX2*, *ISG15*, and *STAT1*—a key mediator of interferon signaling that promotes ISG expression (**Figures 4B, C**). The expression of ISGs peaked at Day 7 under serum-free conditions but gradually declined while remaining significantly higher than in serum-containing cultures through Day 14 (**Figure 4C**).

To better quantify these transcriptional trends, we analyzed published scores for interferon response, cytokine/cytotoxicity, and T cell exhaustion (29, 30). These scores were evaluated at both the single-cell and donor levels. The interferon score was consistently elevated in serum-free cultures, peaking at Day 7 and decreasing thereafter (**Figures 4C, D**). In contrast, the cytokine/cytotoxicity score peaked in serum-supplemented conditions at Day 10, with minimal differences between the two conditions by Day 14 (**Figures 4E, F**). The exhaustion score was transiently higher in serum-free cultures at Day 10 but equalized by the end of the culture (**Figures 4G, H**). Importantly, none of these trends reached statistical significance at the donor level. Taken together, these results indicate that serum-free conditions transiently modulate interferon and cytotoxic gene programs but result in minimal transcriptional differences by the end of γδ T cell expansion.

### 3.5 Serum modulates functional cytokine and degranulation profiles of γδ T cells

**Figure 5:**
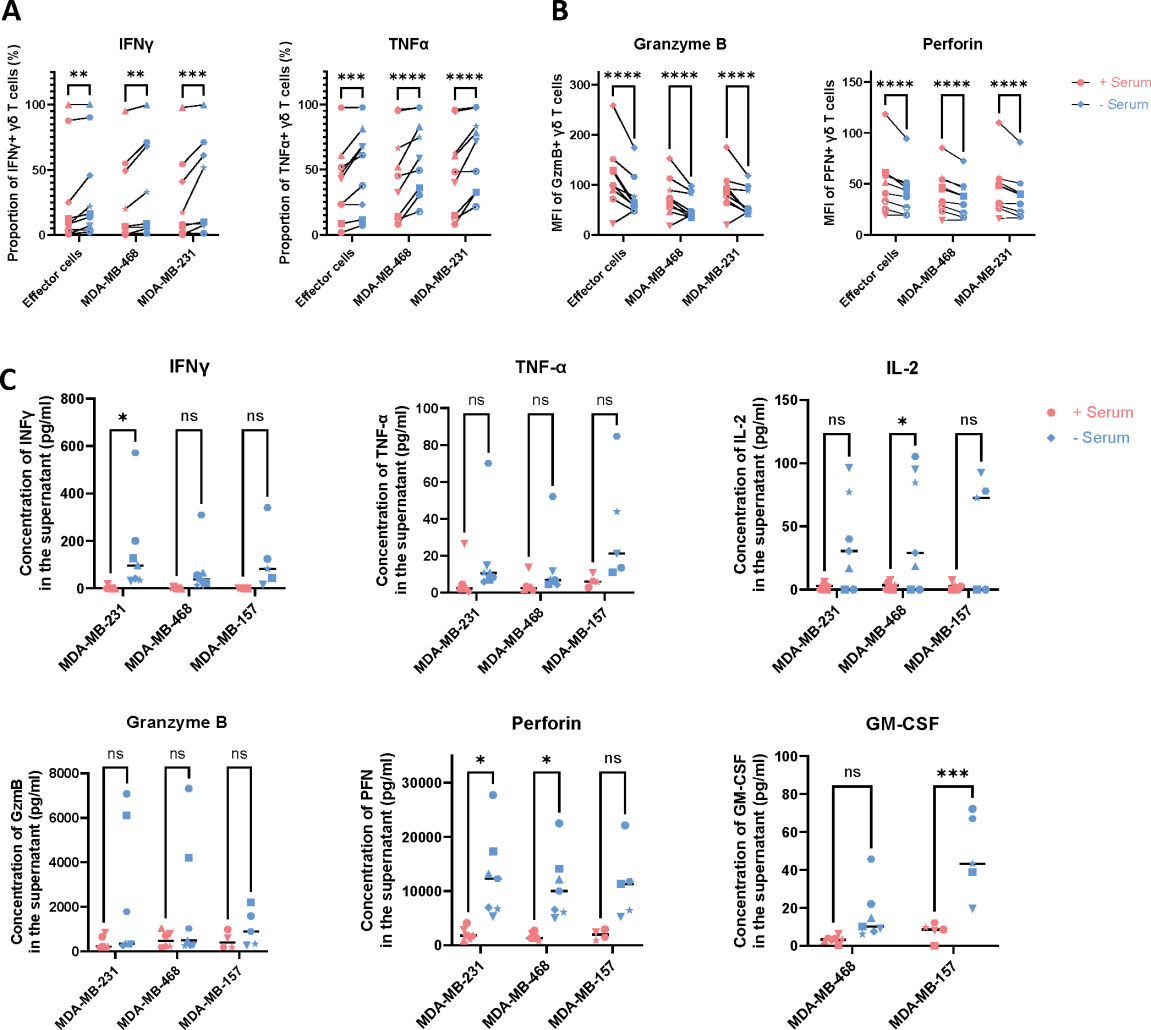
Impact of serum on γδ T cell cytokine and cytotoxic granules production and secretion. **(A, B)** Intracellular cytokine staining of γδ T cells alone (effector cells) or cocultured with the target cells MDA-MB-231 and MDA-MB-468, showing **(A)** the population of IFN-γ+ and TNF-α+ γδ T cells as a proportion of total γδ T cells, and **(B)** the MFI of GzmB+ and PFN+ γδ T cells. Šídák’s multiple comparisons test, n=9. **(C)** Concentrations of cytokines and cytotoxic granules in the supernatant of γδ T cells cocultured with MDA-MB-231, MDA-MB-468, and MDA-MB-157. Šídák’s multiple comparisons test, n=5 for MDA-MB-231 and MDA-MB-468; n=3 for MDA-MB-157. In the figures, the significance levels denoted by stars are as follows: *p < 0.05, **p < 0.01, ***p < 0.001, ****p < 0.0001, ns = non-significant. Different symbols represent different donors.

To validate the scRNA-seq findings, we conducted intracellular cytokine staining and cytokine secretion assays. Following a 2-hours co-culture of γδ T cells with TNBC cell lines, flow cytometry analysis revealed a significant increase in the proportion of γδ T cells producing IFNγ (n=9, p=0.008) and TNFα (n=9, p=0.0003) in serum-free cultured samples compared to those cultivated with serum, even in the absence of target cells. Similar results were observed when γδ T cells were co-cultured with target cells, demonstrating a consistent increase in cytokine production in both conditions (**Figure 5A**). Proportions of GzmB and PFN-positive cells did not differ significantly and approached near 100% across all conditions (data not shown), while their MFI was significantly reduced in serum-free samples, regardless of whether they were incubated alone or with target cells (n=9, p<0.0001 for all conditions) (**Figure 5B**). Supernatants from 24-hour co-cultures of γδ T cells and target cells were analyzed using the MACSPlex T/NK kit to profile cytokine and cytotoxic granule secretion. IFN-γ secretion was significantly elevated in γδ T cells cultured without serum and co-cultured with MDA-MB-231 cells (n=7, p=0.0389), while a non-significant upward trend was observed for the other two TNBC cell lines.

Similarly, perforin secretion increased in serum-free conditions, reaching statistical significance in co-cultures with MDA-MB-231 (n=7, p=0.0483) and MDA-MB-468 cells (n=7, p=0.0346). Although granzyme B (GzmB) and PFN secretion showed non-significant increases across all three target cell lines, IL-2 and GM-CSF followed comparable patterns. Notably, IL-2 secretion was significantly higher in co-cultures with MDA-MB-468 (n=7, p=0.0271), and GM-CSF secretion was significantly elevated in the MDA-MB-157 condition (n=5, p=0.0008) (**Figure 5C**).

### 3.6 Both conditions support broad γδ TCR repertoire without clonal skewing during expansion

We next investigated whether culturing the cells under these distinct conditions would influence the TCR repertoire. We analyzed TCR clonality at the single-cell level in γδ T cells expanded under serum-free and serum-supplemented conditions. Since TCRs were profiled from the same cells used for transcriptome analysis, we could directly link clonal expansion to transcriptional states.

**Figure 6:**
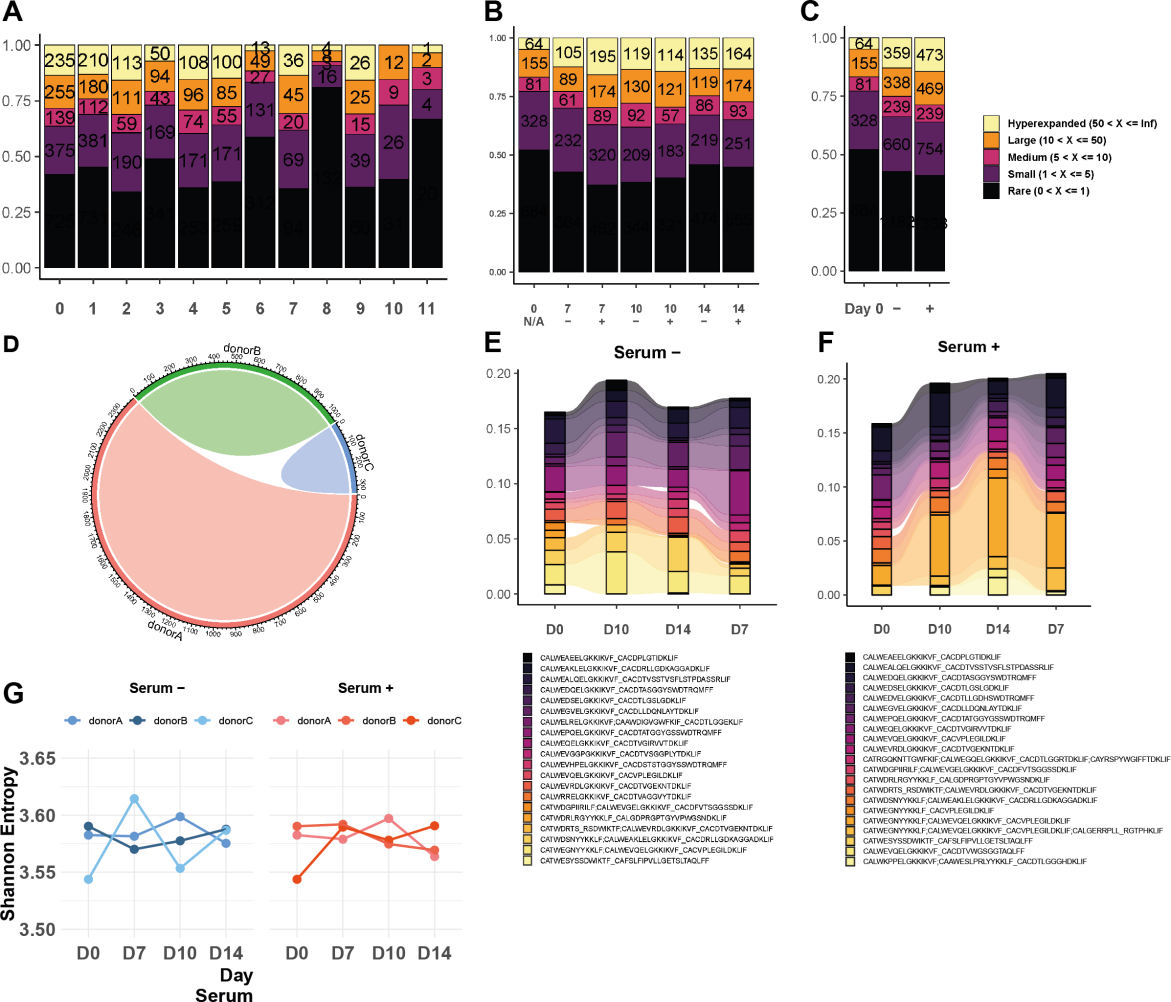
Serum-free culture conditions maintain γδ TCR repertoire diversity during expansion. **(A–C)** Bar plots depict the distribution of clonal sizes across cell clusters, conditions, and days of culture. **(D, E)** Bar plots compare the top 20 most abundant shared clones between different days of culture under serum-free and serum-containing conditions. **(F)** Line graph shows the Shannon diversity score of the γδ TCR repertoire across days of incubation in both serum-free and serum-supplemented conditions, with each point representing an independent donor (n=3).

Clusters 6, 3, and 10—predominantly composed of cells from Day 0—contained a high proportion of singletons and few hyperexpanded clones (**Figure 6A**). When comparing clonal distributions across conditions and time points (**Figures 6B, C**), we observed no consistent differences in the proportion of rare, small, medium, large, or hyperexpanded clones between serum-free and serum-supplemented cultures. Clonotype overlap analysis revealed that each donor harbored a largely private repertoire at baseline (**Figure 6D**). Tracking the top 10 clones across time points (**Figures 6E, F**) showed no evidence of preferential clonal expansion driven by serum supplementation. Finally, Shannon entropy analysis demonstrated stable clonal diversity across both conditions and donors (**Figure 6G**), with no notable divergence between serum-free and serum-containing cultures. Together, these results indicate that both culture conditions support γδ T cell expansion without compromising TCR repertoire diversity or inducing clonal skewing.

### 3.7 Serum re-exposure minimally alters γδ T cell cytotoxicity, degranulation, and cytokine production

**Figure 7:**
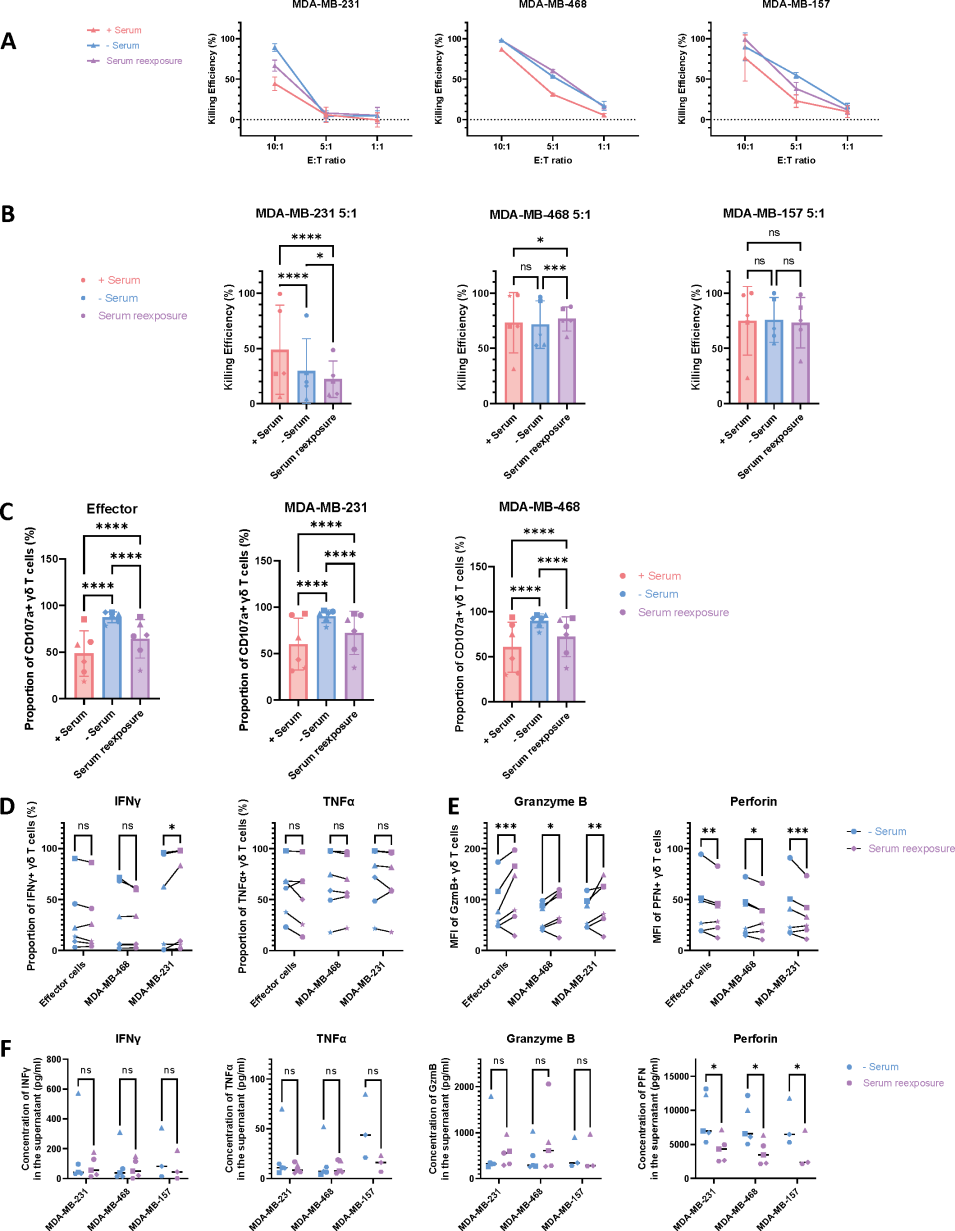
Impact of re-exposure to serum on γδ T cell functionality. γδ T cells expanded without serum were re-exposed to serum in the last two days of culture and then co-cultured with GFP and luciferase-expressing MDA-MB-231, MDA-MB-468, or MDA-MB-157 triple-negative breast cancer cell lines to compare their functionality with cells expanded in serum or cells not re-exposed to serum. **(A)** Luciferase-based assay at different E:T ratios after 24h co-culture. One representative donor. **(B)** Luciferase-based assay at a 5:1 E:T ratio after 24h co-culture. Šídák’s multiple comparisons test, n=5. **(C)** Measurement of CD107a expression on γδ T cells alone (effector cells) or in co-culture for 2h. Šídák’s multiple comparisons test, n=6. **(D, E)** Intracellular cytokine staining of γδ T cells alone (effector cells) or cocultured with the target cells MDA-MB-231 and MDA-MB-468, showing **(D)** the population of IFN-γ+ and TNF-α+ γδ T cells as a proportion of total γδ T cells, and **(E)** the MFI of GzmB+ and PFN+ γδ T cells. Šídák’s multiple comparisons test, n=6. **(F)** Concentrations of cytokines and cytotoxic granules in the supernatant of γδ T cells cocultured with MDA-MB-231 (n=5), MDA-MB-468 (n=5), and MDA-MB-157 (n=3). Šídák’s multiple comparisons test. In the figures, the significance levels denoted by stars are as follows: *p < 0.05, **p < 0.01, ***p < 0.001, ****p < 0.0001, ns = non-significant. Different symbols represent different donors.

In the context of serum-free culture, the cellular product will inevitably be re-exposed to serum upon infusion into the patient. To investigate the impact of serum re-exposure on γδ T cells, cultures were supplemented with human serum during the final 2 days of expansion and compared to cells maintained entirely under serum-free conditions. Cytotoxic activity was assessed using luciferase-based assays at multiple effector-to-target (E:T) ratios following 24 hours of co-culture. The results demonstrated only minor differences in cytotoxicity between serum-re-exposed cells and those continuously expanded in serum-free media across all tested E:T ratios (**Figure 7A**).

At a fixed effector-to-target (E:T) ratio of 5:1, the effects on cytotoxicity varied depending on the target cell line. The cytotoxicity was significantly decreased in the MDA-MB-231 set-up (p=0.0126), while it was significantly increased in the MDA-MB-468 condition (p=0.0001). No significant difference was observed when the γδ T cells were cultivated with MDA-MB-157 (**Figure 7B**, n=5).

Assessment of degranulation capacity, as indicated by CD107a surface expression, revealed a significant decrease in γδ T cells re-exposed to serum compared to those maintained continuously in serum-free conditions (p<0.0001). Nevertheless, CD107a expression in serum-re-exposed cells remained elevated relative to cells expanded in serum-containing media (p<0.0001) (**Figure 7C**, n=6).

Intracellular cytokine staining was performed to evaluate the production of IFN-γ, TNF-α, granzyme B (GzmB), and perforin (PFN) by γδ T cells. No significant differences were observed in the proportions of IFN-γ^+^ and TNF-α^+^ γδ T cells between serum-re-exposed and serum-free conditions, except for a modest increase in IFN-γ production when γδ T cells were re-exposed to serum and co-cultured with MDA-MB-231 cells (p=0.9265) (**Figure 7D**, n=6). Notably, the MFI of GzmB^+^ γδ T cells was significantly elevated across all conditions—whether cultured alone (p=0.0001), with MDA-MB-231 (p=0.0021) or MDA-MB-468 cells (p=0.0108). In contrast, PFN^+^ γδ T cells exhibited a significant decrease in MFI under the same conditions: when cultured alone (p=0.0067), with MDA-MB-231 (p=0.0005) or MDA-MB-468 cells (p=0.0327) (**Figure 7E**, n=6). Analysis of cytokine and cytotoxic granule levels in co-culture supernatants revealed no significant differences between serum-re-exposed and serum-free γδ T cells, except for a reduction in PFN secretion following serum re-exposure, observed in co-cultures with MDA-MB-231 (p=0.0324, n=5), MDA-MB-468 (p=0.0209, n=5) or MDA-MB-157 cells (p=0.0387, n=3) (**Figure 7F**). Collectively, these results indicate that serum re-exposure during the culture period exerts only a limited effect on γδ T cell functionality, including cytotoxicity, degranulation capacity, and cytokine secretion, compared to continuous serum-free expansion.

## 4 Discussion

Given the significant therapeutic potential of γδ T cells in adoptive cell therapy, some protocols have been developed to expand these cells under serum-free conditions (22). However, these approaches often lack a comprehensive analysis of how the presence of human serum in culture environments influence the phenotypic characteristics, functional properties, and overall quality of the expanded γδ T cells. In this study, we investigated the effects of culturing and expanding γδ T cells using both serum-containing and serum-free media. Our work aims to address these aspects by systematically evaluating the impact of serum-free versus serum-containing media on γδ T cell expansion and functionality.

To comprehensively characterize γδ T cells expanded under both culture conditions, we employed scRNA-seq, single-cell T cell receptor sequencing, and different functional *in vitro* assays. Our findings demonstrate that γδ T cells can be effectively expanded in both conditions without compromising the overall yield. The absence of human serum during expansion resulted in higher purity of the γδ T cell product, which is particularly significant for allogeneic applications. γδ T cells expanded under serum-free conditions exhibited a predominantly memory phenotype, characterized by high CD27 expression. This memory phenotype is advantageous for therapeutic applications, as it is associated with improved persistence, enhanced cytokine secretion, and increased cytotoxic potential *in vivo* (31, 32). γδ T cells expanded without serum displayed elevated activation marker expression (CD69, CD56, HLA-DR, and NKG2D) and lower expression of immune checkpoint receptors PD-1 and TIGIT, suggesting a cell product with a robust activation state and potential persistence in the immunosuppressive TME (33, 34).

Although we observed differences in gene expression programs associated with cytotoxicity and interferon signaling between serum-free and serum-supplemented conditions, these were not statistically significant at the donor level and were more pronounced during the mid-phase of the culture but largely minimized by the end of the expansion. These results suggest a transient divergence that converges toward a similar transcriptional state in both conditions. Complementing the transcriptomic data, our TCR analysis demonstrated that γδ T cells maintained a highly diverse and polyclonal repertoire during expansion in both serum-free and serum-supplemented conditions. We did not observe any significant differences in the clonal size distribution, expansion dynamics of dominant clones, or overall repertoire diversity as measured by Shannon entropy. Notably, serum-free culture did not preferentially promote expansion of specific clones, indicating an unbiased expansion environment. This preservation of TCR diversity is critical for maximizing the therapeutic breadth of γδ T cells in targeting heterogeneous tumor antigens.

γδ T cells expanded in serum-free conditions exhibited similar or even enhanced cytotoxic activity against TNBC cell lines compared to those expanded in serum-containing media. This was evident across various E:T ratios and supported by robust degranulation activity, as indicated by CD107a expression. Notably, while intracellular cytokine staining revealed lower intracellular levels of granzyme B and perforin in serum free-expanded cells, and scRNA-seq showed that they had a lower cytotoxicity score, an increased secretion of these cytotoxic mediators was observed in the MACSPlex assay. The discrepancy between intracellular levels and secretion suggests that γδ T cells expanded in serum-free medium are highly active, releasing granules efficiently upon encountering target cells. Moreover, γδ T cells expanded without serum demonstrated significantly increased secretion of IFNγ, a key cytokine in anti-tumor immunity. Similar trends of enhanced IFNγ production have been observed in serum-free αβ T cells (16, 35). This is particularly relevant, as IFNγ plays a crucial role in modulating the tumor microenvironment. It promotes the upregulation of MHC-I and MHC-II molecules on tumor cells, enhancing their visibility to immune cells, and facilitates the recruitment and activation of other immune effector cells, such as macrophages and cytotoxic lymphocytes (36). These findings suggest that serum-free γδ T cells may retain cytotoxic capabilities and could actively contribute to reshaping the immunosuppressive tumor microenvironment, further amplifying their therapeutic potential.

When γδ T cells expanded in serum-free conditions were re-exposed to serum, their characteristics began to partially resemble those of γδ T cells that were expanded with serum. This observation suggests that specific serum components might exert both activating and inhibitory effects on γδ T cells. While the exact factors responsible for these effects remain unclear, this suggests that human serum can impact γδ T cell function, even at later stages of culture. Identifying which factors are beneficial for γδ T cell function will be crucial to develop serum-free manufacturing processes that can support the expansion of large numbers of γδ T cells. Interestingly, some alternatives, including human platelet lysate (hPL), Physiologix™ xeno-free hGFC, and other chemically-defined supplements have been shown to support immune cell expansion in serum-free conditions (37–39). Along these lines, the use of human serum albumin in thawing medium has been shown to enhance γδ T cell viability during cryopreservation, further highlighting the importance of optimizing culture conditions for clinical applications (40).

On top of revealing crucial differences between γδ T cells expanded with and without serum, scRNA-seq proved useful to gain a deeper insight into the manufacturing process. For instance, γδ T cells did not show further proliferation when they reached 14 days of expansion, suggesting that the cells may have entered a quiescent or terminal differentiation state. Furthermore, transcriptomic data at day 14 revealed stabilization of activation-associated gene programs and no significant upregulation of exhaustion markers, suggesting that extending culture beyond this point may not only be inefficient but could also risk phenotypic deterioration. Thus, day 14 represents a plateau phase where optimal yield and functionality converge, maintaining functional potential while avoiding unnecessary extended culture. These findings have direct implications for production processes, enabling a more efficient and cost-effective approach by eliminating redundant culture time and reducing exposure to stress signals that may impair cell quality.

Our study demonstrates the successful expansion of functional γδ T cells in serum-free conditions, maintaining their phenotype while enhancing purity, activation, and anti-tumor reactivity. It also highlights the utility of scRNA-seq in uncovering the nuanced effects of human serum on γδ T cell characteristics, revealing both activating and inhibitory impacts on their function and phenotype. Serum re-exposure experiments further emphasized the complex role of serum components, underscoring the need for deeper investigation into specific factors driving these effects. These findings pave the way for leveraging scRNA-seq to optimize platforms for γδ T cell therapies, enhancing consistency and efficacy in clinical applications.

By investigating optimal expansion conditions that preserve γδ T cell functionality, this study contributes to the development of scalable and consistent manufacturing protocols designed to meet the needs of clinical applications.

### 4.1 Limitations of this study

While our study aims to provide a comprehensive analysis of the ex-vivo expansion of γδ T cells with and without human serum, certain limitations should be considered when interpreting the findings. Our single-cell dataset was derived from healthy donors and comprised a relatively small sample size. Furthermore, functional characterization was restricted to *in-vitro* cytotoxicity assays against TNBC cell lines, which were chosen because they are well established and extensively studied in our laboratory. However, *in-vivo* validation is still lacking, which would be necessary to fully capture the potential of γδ T cell responses within a complex physiological environment. Finally, although this study focuses on small-scale γδ T-cell expansion, it does not include assessments of scalability under serum-free conditions using closed or automated manufacturing systems, an aspect that can be addressed in future studies.

## Data Availability

Sequencing data (FASTQ files) are available upon request to S. The processed data reported in this paper is available to download from https://doi.org/10.5281/zenodo.17590095. Codes to reproduce the data analysis and figures are available at the same repository.
