# Supplementary material for: Human serum influences functional plasticity and transcriptomic landscape of γδ T cells in vitro
Source: Front Immunol. 2026 Feb 10;17:1722590. doi: 10.3389/fimmu.2026.1722590 (PMC12929466; doi:10.3389/fimmu.2026.1722590)
Supplement: Supplementary file 1 [file Table1.docx]

**Supplementary Data**

**Supplementary Figure 1**

Flow cytometry panels

|  | | **Cell composition** | **Phenotype** | **Subsets** | **Inhibitory** |
| --- | --- | --- | --- | --- | --- |
| **V1** | **VioBlue** | CD3 | δ1 | δ1 | δ1 |
| **V2** | **VioGreen** |  | HLA-DR |  | KIR2D |
| **B1** | **FITC** |  | CD45RA | γ9 |  |
|  | **VioBright B515** | CD56 |  |  | TIM3 |
| **B2** | **PE** | PD-1 | CD27 | NKG2D | PD-1 |
| **B3** | **PerCP** | 7-AAD | 7-AAD | 7-AAD | 7-AAD |
| **B4** | **PE-Vio 770** | CD14 | CD69 |  | TIGIT |
| **R1** | **APC** | γδ | γδ | γδ | γδ |
| **R2** | **APC-Vio 770** | CD19 | δ2 | δ2 | δ2 |

**Supplementary Figure 2**

Flow cytometry gating strategies for each panel.

Panel Cell Composition

**
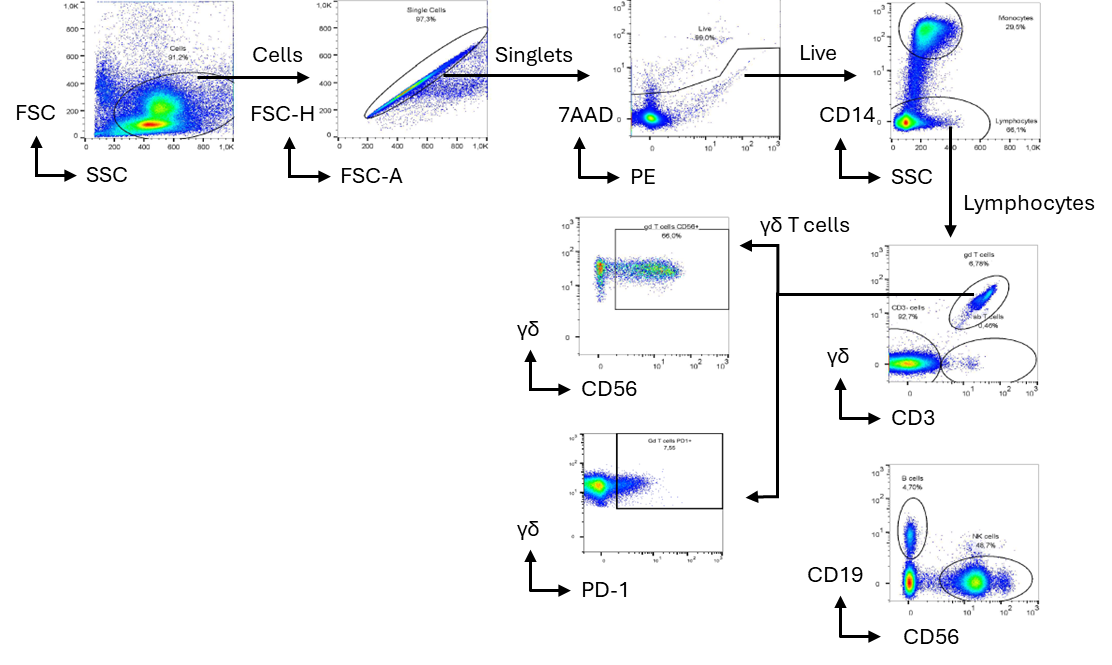
**

Panel Cell Phenotype


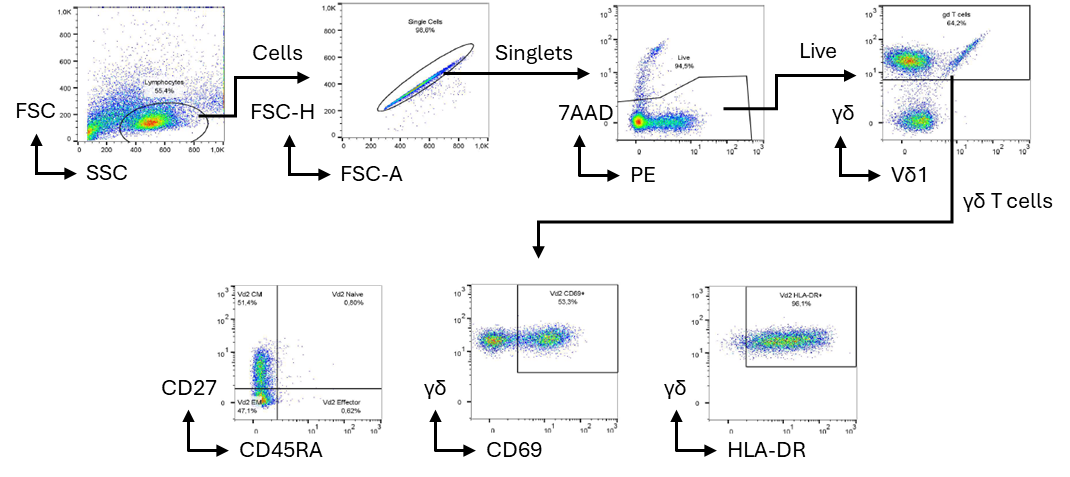


Panel Exhaustion


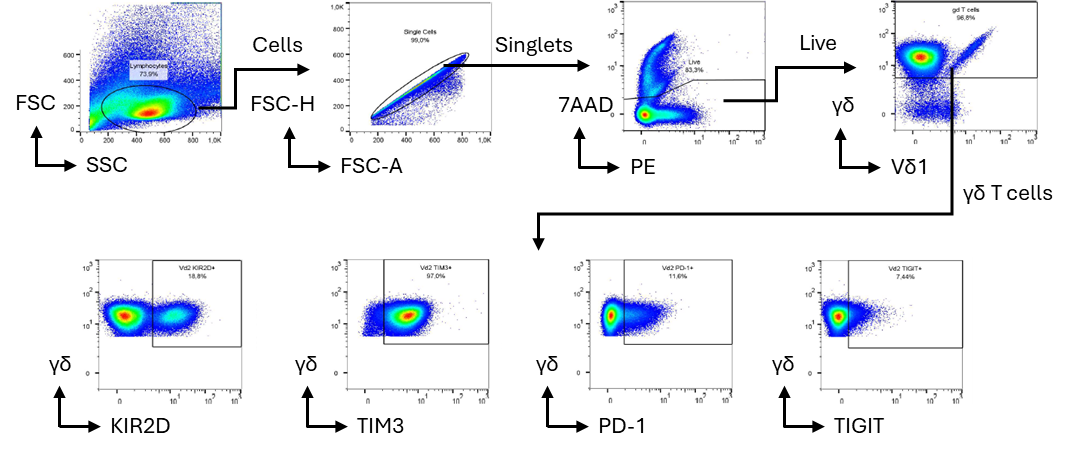


Panel Subsets


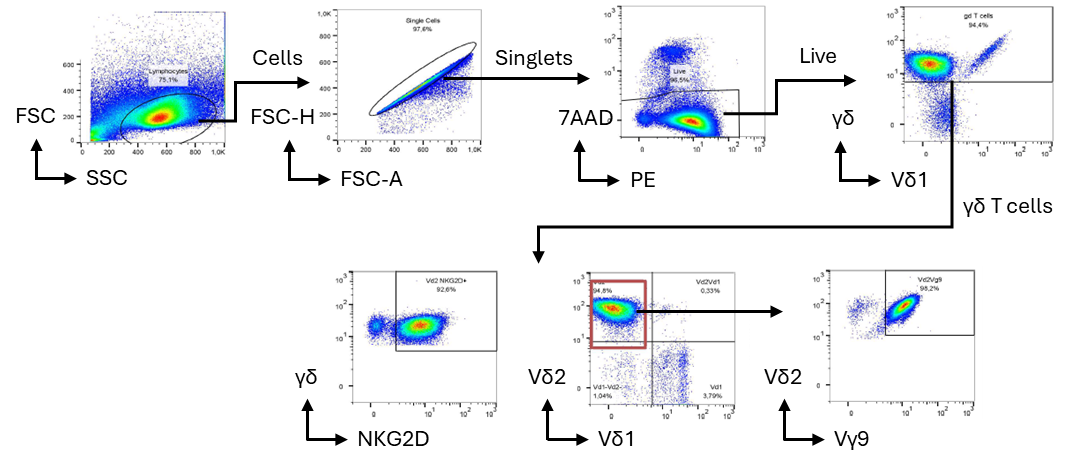


**Supplementary Figure 3**

|  | **Hashing antibody** | **Barcode** | **Reaction** |
| --- | --- | --- | --- |
| donorA, day 0 | Separate reaction on another day (no hashtaging) | | #1 |
| donorA, day 7 -S | TotalSeq™-C0252 anti-human Hashtag 2 Antibody | TGATGGCCTATTGGG | #1 |
| donorA, day 10 -S | TotalSeq™-C0253 anti-human Hashtag 3 Antibody | TTCCGCCTCTCTTTG |  |
| donorA, day 14 -S | TotalSeq™-C0254 anti-human Hashtag 4 Antibody | AGTAAGTTCAGCGTA |  |
| donorA, day 7 +S | TotalSeq™-C0255 anti-human Hashtag 5 Antibody | AAGTATCGTTTCGCA |  |
| donorA, day 10 +S | TotalSeq™-C0256 anti-human Hashtag 6 Antibody | GGTTGCCAGATGTCA |  |
| donorA, day 14 +S | TotalSeq™-C0257 anti-human Hashtag 7 Antibody | TGTCTTTCCTGCCAG |  |
| donorB, day 0 | TotalSeq™-C0251 anti-human Hashtag 1 Antibody | GTCAACTCTTTAGCG | #2 |
| donorB, day 7 -S | TotalSeq™-C0252 anti-human Hashtag 2 Antibody | TGATGGCCTATTGGG |  |
| donorB, day 10 -S | TotalSeq™-C0253 anti-human Hashtag 3 Antibody | TTCCGCCTCTCTTTG |  |
| donorB, day 14 -S | TotalSeq™-C0254 anti-human Hashtag 4 Antibody | AGTAAGTTCAGCGTA |  |
| donorB, day 7 +S | TotalSeq™-C0255 anti-human Hashtag 5 Antibody | AAGTATCGTTTCGCA |  |
| donorB, day 10 +S | TotalSeq™-C0256 anti-human Hashtag 6 Antibody | GGTTGCCAGATGTCA |  |
| donorB, day 14 +S | TotalSeq™-C0257 anti-human Hashtag 7 Antibody | TGTCTTTCCTGCCAG |  |
| donorC, day 0 | TotalSeq™-C0251 anti-human Hashtag 1 Antibody | GTCAACTCTTTAGCG | #3 |
| donorC, day 7 -S | TotalSeq™-C0252 anti-human Hashtag 2 Antibody | TGATGGCCTATTGGG |  |
| donorC, day 10 -S | TotalSeq™-C0253 anti-human Hashtag 3 Antibody | TTCCGCCTCTCTTTG |  |
| donorC, day 14 -S | TotalSeq™-C0254 anti-human Hashtag 4 Antibody | AGTAAGTTCAGCGTA |  |
| donorC, day 7 +S | TotalSeq™-C0255 anti-human Hashtag 5 Antibody | AAGTATCGTTTCGCA |  |
| donorC, day 10 +S | TotalSeq™-C0256 anti-human Hashtag 6 Antibody | GGTTGCCAGATGTCA |  |
| donorC, day 14 +S | TotalSeq™-C0257 anti-human Hashtag 7 Antibody | TGTCTTTCCTGCCAG |  |

**Table listing hashing antibodies used to multiplex the samples.**

**Supplementary Figure 4**

Cellular composition assessed by flow cytometry after expansion: (A) Vδ2 population and (B) NK cell population. Paired Wilcoxon comparisons test, n=12. ***p < 0.001.

**Supplementary Figure 5**

Cellular viability assessed by flow cytometry after expansion. Paired Wilcoxon comparisons test, n=12. ns=non-significant.

**Supplementary Figure 6**

γδ T cell phenotype determined by flow cytometry based on CD27 and CD45RA expression. Paired Šídák's multiple comparisons test, n=12. *p < 0.05, ns = non-significant.

**Supplementary Figure 7**

|  | **+ Serum** | **- Serum** |
| --- | --- | --- |
| CD69 (n=15) | 67.49 [57.36 – 77.62] | 81.20 [74.97 – 87.43] |
| CD56 (n=15) | 68.46 [61.04 – 75.88] | 86.08 [80.57 – 91.59] |
| HLA-DR (n=15) | 88.71 [82.65 – 94.77] | 97.61 [96.66 – 98.57] |
| NKG2D (n=6) | 78.87 [67.41 – 90.33] | 93.08 [84.91 – 101.3] |
| KIR2D (n=6) | 20.44 [9.245 – 31.63] | 16.28 [10.49 – 22.08] |
| PD-1 (n=6) | 34.23 [19.61 – 48.86] | 9.510 [-1.73 – 20.75] |
| TIM3 (n=6) | 90.90 [82.14 – 99.66] | 94.45 [90.31 – 98.59] |
| TIGIT (n=6) | 18.24 [8.64 – 27.83] | 7.08 [2.80 – 11.37] |

Mean and confidence intervals of activation and inhibitory/exhaustion markers determined by flow cytometry after expansion.

**Supplementary Figure 8**


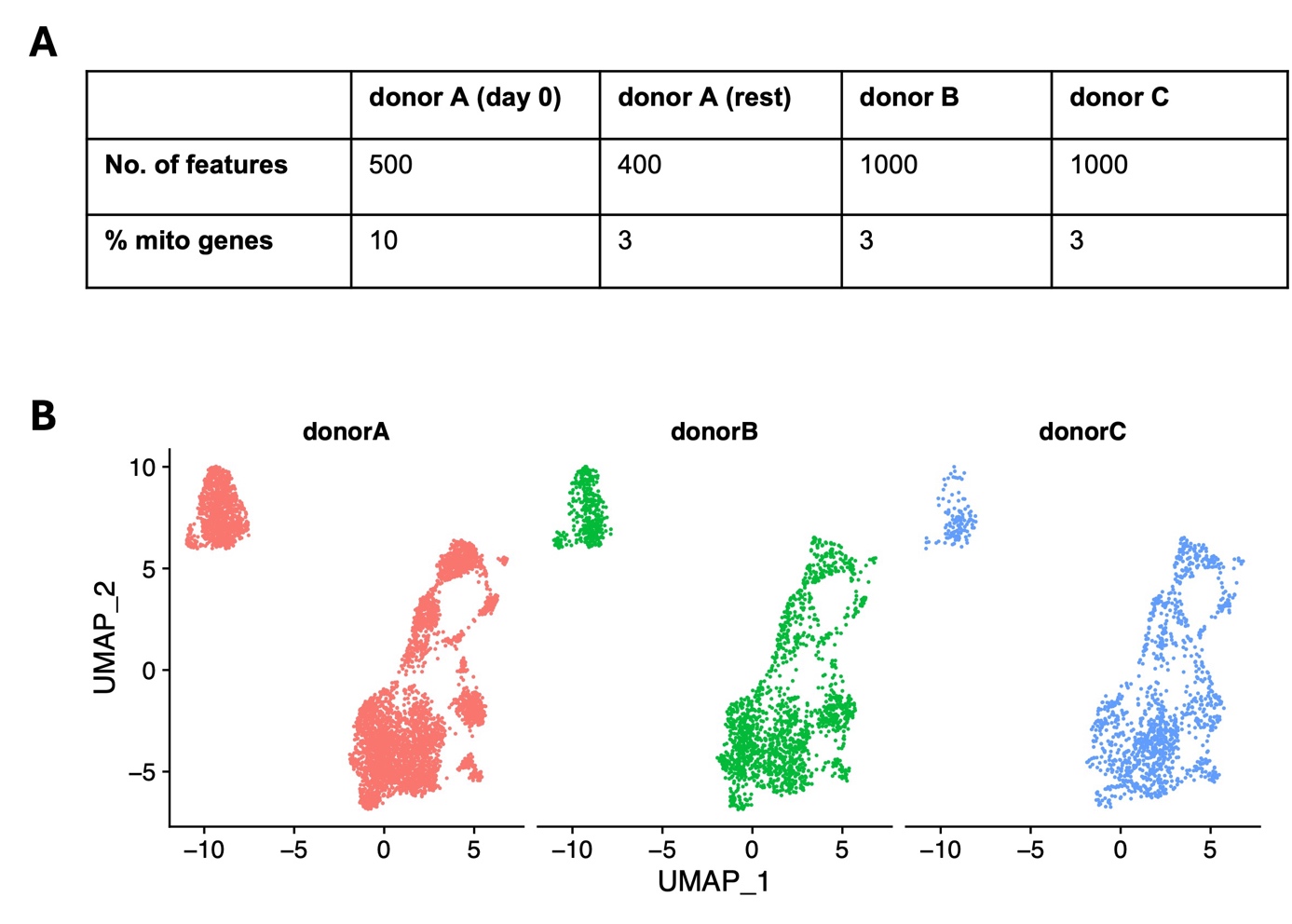


Quality control analysis of the scRNA-seq data. (A) Table listing the cut-offs used to remove low-quality cells for scRNA-seq analysis. (B) UMAP representation showing the origin of cells across different donors in different colors.
